# Advanced assessment of migration and invasion of cancer cells in response to mifepristone therapy using double fluorescence cytochemical labeling

**DOI:** 10.1186/s12885-019-5587-3

**Published:** 2019-04-24

**Authors:** Sabrina J. Ritch, BreeAnn N. Brandhagen, Alicia A. Goyeneche, Carlos M. Telleria

**Affiliations:** 1 Experimental Pathology Unit, Department of Pathology, Faculty of Medicine, McGill University, 3775 University Street, Montreal, Qc H3A 2B4 Canada; 2 Present address: Research Acceleration Office, 2001 Campus Delivery, University Services Center, Colorado State University, Fort Collins, CO 80523 USA

**Keywords:** Fluorescence cytochemical labeling, Mifepristone, Metastasis, Cell migration, Cell invasion, Fibrillar actin, DNA

## Abstract

**Background:**

Previous work in our laboratory demonstrated that antiprogestin mifepristone impairs the growth and adhesion of highly metastatic cancer cells, and causes changes in their cellular morphology. In this study, we further assess the anti-metastatic properties of mifepristone, by studying whether cytostatic doses of the drug can inhibit the migration and invasion of various cancer cell lines using a double fluorescence cytochemical labeling approach.

**Methods:**

Cell lines representing cancers of the ovary (SKOV-3), breast (MDA-MB-231), glia (U87MG), or prostate (LNCaP) were treated with cytostatic concentrations of mifepristone. Wound healing and Boyden chamber assays were utilized to study cellular migration. To study cellular invasion, the Boyden chamber assay was prepared by adding a layer of extracellular matrix over the polycarbonate membrane. We enhanced the assays with the addition of double fluorescence cytochemical staining for fibrillar actin (F-actin) and DNA to observe the patterns of cytoskeletal distribution and nuclear positioning while cells migrate and invade.

**Results:**

When exposed to cytostatic concentrations of mifepristone, all cancer cells lines demonstrated a decrease in both migration and invasion capacities measured using standard approaches. Double fluorescence cytochemical labeling validated that mifepristone-treated cancer cells exhibit reduced migration and invasion, and allowed to unveil a distinct migration pattern among the different cell lines, different arrays of nuclear localization during migration, and apparent redistribution of F-actin to the nucleus.

**Conclusion:**

This study reports that antiprogestin mifepristone inhibits migration and invasion of highly metastatic cancer cell lines, and that double fluorescence cytochemical labeling increases the value of well-known approaches to study cell movement.

**Electronic supplementary material:**

The online version of this article (10.1186/s12885-019-5587-3) contains supplementary material, which is available to authorized users.

## Background

Cancer metastasis and burden of secondary tumors are the most common causes of mortality for many patients, accounting for nearly 90% of cancer-related deaths [1]. One key aspect of metastasis is the invasive capacity of the cells, which is mainly driven by cell motility [2]. Cancer cell motility, in turn, is heavily dependent on changes in tumor cell morphology caused by dynamic modifications in the polymerization of actin leading to rearrangements of the cytoskeleton [3]. The changes in cellular morphology and their impact on motility are associated with changes among epithelial and mesenchymal phenotypes, a process known as epithelial-to-mesenchymal transition (EMT). The transition from an epithelial to a more mesenchymal state is linked to morphological modifications, loss of tight junctions, remodeling of the cytoskeleton, and acquisition of migratory and invasive capacities [4]. Such migratory and invasive capacities are commonly assessed by a variety of experimental approaches which have been amply described [5].

The rationale for classical anti-cancer therapy has long been to target cell proliferation at the primary site without, unfortunately, discriminating cancer cells from normal cycling cells [6]. This approach was improved by the introduction of targeted therapies and immunotherapies that brought about reduced toxicities as they target cancer cells while sparing normal cells [7, 8]. However, to treat cancer more effectively, we should further focus on preventing the formation and growth of metastatic carcinoma cells. We should consider that inhibition of migration, associated with the process of metastasis, might be as important as inhibition of cell proliferation. Agents that negatively influence *both* mechanisms might provide a novel tool to fight cancer, in particular if they inhibit cell proliferation at the sites of metastasis while preventing migration of such cells to new niches.

Previous work in our laboratory has shown that the prototypical member of the family of antiprogestins, mifepristone (MF), can efficiently inhibit the growth of cancer cells of ovarian, breast, prostate, and glial origin, all known for their high metastatic potential [9]. We demonstrated that the anti-cancer effect of MF does not require the presence of progesterone receptors [9], involves cell cycle arrest at the G1 phase of the cell cycle associated with the inhibition of cyclin-dependent kinase Cdk2 [10, 11], and triggers cellular stress and autophagy, making it useful in combination therapies with proteasome inhibitors and autophagy blockers [12]. Furthermore, we provided evidence that MF interferes with the adhesive capacity of cancer cells by altering fibrillar actin (F-actin) distribution and promoting the formation of membrane ruffling [13], represented by sheet-like membrane protrusions devoid of adhesive properties [14].

In this work, we demonstrated that MF inhibits migration and invasion via standard approaches, and validated and enhanced a method for visualizing migratory and invasive cells upon double fluorescence cytochemical staining. Labeling migratory and invasive cells with a fluorescent probe linked to Phalloidin allowed us to observe the changes in F-actin distribution while cells are migrating and invading. Furthermore, the simultaneous labeling of the nucleus with a second fluorescent agent capable of binding DNA added further detail to visualize the position of the nucleus relative to the cytoplasm while cells are migrating and invading. The double fluorescence cytochemical labeling increases the value of two well-known approaches—the wound healing and Boyden chamber assays—to study cell movement by simple staining, increasing the level of detail of cell morphology in motion.

## Methods

### Cell culture

The carcinoma cell lines were all obtained from the American Type Culture Collection (ATCC, Manassas, VA). SKOV-3 (ovarian cancer; Cat. HTB-77) were obtained in 2003 (lot 1659235). U87MG (gliobastoma, Cat. HTB-14), MDA-MB-231 (breast cancer, Cat. HTB-26), and LNCaP (prostate cancer, Cat. CRL-1740), were all obtained in 2008 (lots 5105357, 57618051, and 7658493, respectively). All cell lines were cultured as previously described in detail [9].

### Wound healing assays

**Fig. 1:**
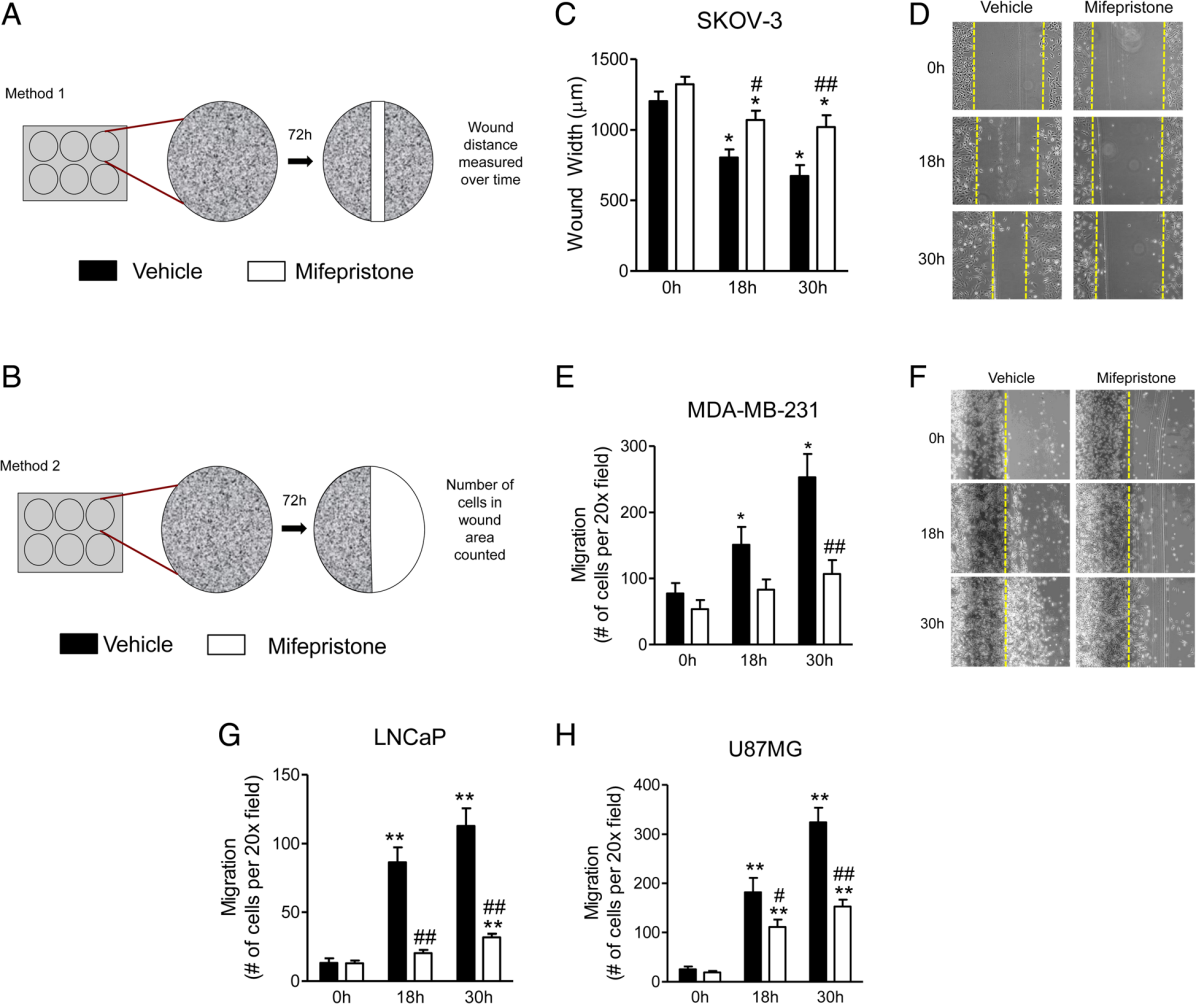
Mifepristone impairs the migration of cancer cells as shown in scratch assays. The schematics of the two methods used to study migration via a wound healing approach are shown in panels (**a**) and (**b**). In (**c**) and (**d**) SKOV-3 cells were treated with vehicle or 23.5 μM MF for 72 h prior to wounding. Multiple images were taken of each well at various time-points. The width of the wound was measured three times per image. Data shown represent the mean ± s.e.m. of three independent experiments completed in triplicate. Vehicle (closed bars), MF (open bars). *P < 0.01 vs. 0 h; #*P* < 0.05 vs. vehicle; ## *P* < 0.01 vs. vehicle. Statistical analysis of individual treatment groups over time (vs. time = 0 h) was done using a one-way ANOVA followed by Tuckey’s multiple-comparison test. Analysis between treatment groups over time (vs. vehicle) was performed using a two-way ANOVA followed by Bonferroni’s multiple- comparison test. Panels (**e**) and (**f**) show the migration of MDA-MB-231 cells upon treatment with 30 μM MF for 72 h prior to wounding. Notice that the dark line detected by the microscope to the left of the border of the wounded area, denoted in yellow, represents the original line traced with a pen underneath the plate before the wound was created. This artifact does not preclude us for counting the cells that migrated away of the wound. Similar migratory experiments in response to 20 μM MF for 72 h were done using LNCaP (**g**) and U87MG (**h**) cells. The statistical analysis done in **e**, **g**, and **h** is similar to that described for **c**

For all cell lines studied, the concentrations of mifepristone used (MF; Corcept Therapeutics, Menlo Park, CA) were those that were previously demonstrated to cause inhibition of cell growth by inducing cytostasis, without triggering cell death (i.e. lethality) [13] (Table 1). In a first approach (Fig. 1a), SKOV-3 cells were seeded in 6-well plates at a density of 100,000 cells/well. Cells were allowed 24 h to attach in the presence of 10% fetal bovine serum (FBS, Atlanta Biologicals, Lawrenceville, GA) containing culture media as previously described [9]. The media was then removed and replaced with 5% FBS-containing media with vehicle [dimethyl sulfoxide (DMSO); Mediatech, Inc., Manassas, VA], or MF for 72 h. After treatment, a wound or denuded region was created with a p20 pipette tip in a consistent angle and using consistent pressure following recommended standard operating procedures [15]. The cells were washed three times with phosphate buffered saline (PBS; Mediatech) to remove detached cells. The PBS was removed and replaced with 5% FBS-containing media with vehicle or MF. Migration of cells was monitored for 18 and 30 h. Multiple images were taken of each well using a Zeiss Axiovert M200 inverted phase-contrast microscope (Carl Zeiss, Thornwood, NY). The width of the wound was then measured using tools of the AxioVision program (Carl Zeiss).

**Table 1:** Concentrations of MF used to cause growth arrest without inducing lethality of the cell lines studied and their doubling times (DT)

Cell line	Tissue type	DT (h)	MF (μM)
SKOV-3	Ovary	36.9	23.5
MDA-MB-231	Breast	33.3	30
LNCaP	Prostate	50.7	20
U87MG	Glia	36.9	20

Data selected from our previous published works [9, 13]

A second approach was used for U87MG, LNCaP, and MDA-MB-231 cells, because we encountered difficulties in clearly assessing cell migration into the wound using phase-contrast microscopy: these cells do not retain intercellular junctions entirely, having the tendency to move more individually rather than in sheets. Thus, we developed a modified scratch assay in which, instead of wounding a selected segment with a pipette tip, we removed the entire half of the cells in the otherwise confluent cell culture. Thus, we were able to count migrating cells into the clear area (Fig. 1b) [5, 16]. Cells were plated as described above, and treatment with MF was performed for 72 h before wounding. A reference line was then drawn on the bottom of each well, effectively separating the well into two halves. A sterile cotton swab was then used to remove all cells on the right of the reference line (Fig. 1b). The wells were washed three times with PBS to remove all detached cells and debris. The PBS was removed and replaced with the appropriate 10% FBS-containing media with vehicle or MF. Migration of cells was recorded using a Zeiss Axiovert M200 inverted phase-contrast microscope. Cells moving into the open area to the right of the reference line were then counted.

To validate the double fluorescence staining method, we were able to perform the same wound healing experiments using approach one (Fig. 1a) for all cell lines with the addition of Alexa Fluor® 594 Phalloidin followed by DAPI labeling to improve the visualization of cytoplasm and nuclear morphologies. Cells, fixed with 4% paraformaldehyde (PFA; Sigma Chemical Co., St. Louis, MO), were permeabilized using 0.1% Triton X-100 (Sigma) for 5 min. To reduce background staining, cells were then incubated with PBS containing 1% bovine serum albumin (BSA; Wisent Bioproducts, St. Bruno, QC) for 20 min. The stock solution of Alexa Fluor® 594 Phalloidin (Life Technologies, Carlsbad, CA) was diluted from its 6.6 μM at a 1:40 ratio, in PBS containing 1% BSA (5 μL stock solution in 200 μL PBS). Cells were incubated with diluted Alexa Fluor® 594 Phalloidin for 20 min. The 5 mg/mL stock solution of DAPI (Life Technologies, Carlsbad, CA) was diluted to a final working concentration of 300 nM, in PBS containing 1% BSA. Cells were incubated with diluted DAPI solution for 10 min. As the visibility of individual cells was enhanced by the contrast provided by fluorescence, we were able to assess the cells migrating into the wounded area with sufficient precision, despite the different movement modalities encountered among the four cell lines. Using a Leica DMi8 inverted fluorescence microscope and a Leica LAS X software (Leica Microsystems Canada, Concord, ON), migratory cells going into the wound were imaged to visualize wound closure and migration patterns of each cell line.

### Filter-based assays

#### Migration

Cells were treated in cell culture flasks for 72 h with either regular media or MF-containing media at the concentrations previously selected to be cytostatic for each cell line (Table 1). The cells were then trypsinized and resuspended in serum-free media containing vehicle or MF, and plated in the wells containing the insert chambers at a density of 50,000 cells per well. A 10% FBS-containing media was then added to the lower chamber to serve as a chemoattractant. Cells were allowed 9, 18, or 24 h to migrate across the 8 μm polycarbonate membrane, at which point the cells were fixed in 100% methanol (Fisher Scientific, Fair Lawn, NJ) for 30 min. Using a sterile cotton swab, the non-migratory cells remaining in the upper chamber were removed. The inserts were then stored in PBS at 4 °C. The membranes and attached cells were stained with 0.25% crystal violet (Sigma) for 20 min. Inserts were rinsed multiple times with PBS. The migratory cells were counted colorimetrically with a Nikon Diaphot inverted microscope (Nikon, Garden City, NY) using an average of nine 20x fields per insert.

#### Invasion

To study the invasion of cancer cells using the Boyden chamber inserts, cells were grown and treated as described in the migration section. However, before plating, the inserts were coated with a layer of extracellular matrix (ECM) gel from Engelbreth-Holm-Swarm murine sarcoma (Sigma). The stock ECM gel (9.1 mg/ml) was thawed overnight at 4 °C and then diluted in cold serum-free media to a working amount of 60 μg per insert. Each insert was coated with 100 μl of diluted ECM gel and incubated overnight at 37 °C in a humidified atmosphere in the presence of 5% CO2. Following incubation of the gel layer, cells were plated at the same density and in the same manner as described in the migration section. After allowing 18 or 24 h for invasion, cells were fixed, stained with 0.25% crystal violet, and quantified as previously described.

#### Visualization of migrated cells using cytochemical double fluorescence staining

Both migration and invasion assays were repeated with the addition of a double fluorescent staining consisting of Alexa Fluor®-594 Phalloidin and SYTOX® Green (Molecular Probes, Eugene, OR). As larger Boyden chamber plates were used (6-well plates), cells were seeded at a density of 200,000 cells per well and were left to migrate or invade (if inserts were pre-coated with ECM gel) for 6 or 24 h. Migrated cells were then fixed with 4% PFA for 20 min. The fixed cells were first permeabilized using 0.1% Triton X-100 in PBS for 5 min at room temperature, after which they were pre-incubated with PBS containing 1% BSA for 20 min in order to reduce background staining. Cells were incubated with the diluted Alexa Fluor® 594 Phalloidin for 20 min. During the last 10 min of incubation, 4 μl of a 50 μM dilution of SYTOX® Green Nucleic Acid Stain (5 mM stock solution in DMSO) was added to each well. After incubation, cells were washed with PBS and stored in PBS at 4 °C. Using the Leica DMi8 inverted fluorescence microscope and the Leica LAS X software, twenty 20x field images were taken of each well and migration or invasion were measured as the average number of cells per 20x field.

### Immunocytochemistry of phospho-histone H3

Cells having undergone a wound healing assay for 24 h were washed with PBS, and fixed with 4% PFA for 20 min. Cells were permeabilized with 0.1% Triton X-100 for 5 min and, to reduce background staining, they were incubated with PBS containing 1% BSA for 20 min. A 0.5 mg/mL stock solution of rabbit monoclonal phospho-histone H3 (pHH3) antibody (Cat# 06–570, Millipore Sigma, Burlington, MA) was diluted to a working concentration of 2 μg/mL, and cells were incubated with the primary antibody overnight. The next morning, cells were washed three times with PBS. A 2 mg/mL stock solution of goat anti-rabbit IgG Alexa Fluor® 488 secondary antibody (Cat# A11034, Life Technologies, Carlsbad, CA) was diluted to a working concentration of 2 μg/mL, and cells were incubated with the antibody for 30 min. After the incubation, cells were washed with PBS and stored in PBS at 4 °C. Images were taken using the Leica DMi8 inverted fluorescence microscope and the Leica LAS X software.

### Assessment of morphology using cytochemical double fluorescence staining upon treatment with mifepristone

Cells were seeded in 6-well culture plates, at a density of 50,000 or 100,000 cells per well. Cells were allowed to attach overnight in regular media. After attachment, the cells were either treated with DMSO or with various concentrations of MF for 72 h. At the end of the incubation, plates were washed with PBS, and fixed with 4% PFA for 20 min, after which they were stored in PBS at 4 °C until processed for fluorescence staining as previously described. Images were taken by fluorescence microscopy with a Zeiss Axiovert M200.

### Statistical analysis

All data are represented as means ± s.e.m. and statistical significance was consistently defined as *p* < 0.05. One-Way ANOVA followed by Tukey’s multiple comparison test, or two-way ANOVA followed by Bonferroni’s multiple comparison test were used as appropriate. Experiments were repeated three times in triplicates.

## Results

### Mifepristone alters the morphology of cancer cells in a dose-related manner

We previously observed, using phase contrast microscopy, that cytostatic concentrations of MF cause elongation of the cytoplasm in various cancer cell types [13]. Now, double fluorescence labeling allows to simultaneously appreciate that both, morphological changes and reduced cellular density, induced by MF, are dose-related (Additional file 1: Figure S1A-D). The double fluorescence staining emphasizes the position of the nucleus within the cell while highlighting the pronounced cytoplasmic stretching along all the cell lines.

### Mifepristone attenuates migration and invasion of cancer cells

Cancer-cell migration is critical for distant metastases. The cell’s ability to rearrange its cytoskeleton and propel itself forward is necessary before invasion and movement throughout various tissues can occur. Cells that cannot move towards a source of nutrition (blood vessels, for instance) will likely not survive. Thus, due to the importance of this process, we studied the impact of morphological changes triggered by cytostatic concentrations of MF [13] (Table 1) on cellular migration.

The migration of SKOV-3 cells in a wound healing assay was impaired at both 18 and 30-h time-points. At 30 h, vehicle-treated cells had substantially closed the wound, whereas MF-treated cells were unable to move any farther than they had after 18 h, leaving the wound relatively at the same initial size (Fig. 1c, d).

In MDA-MB-231 cells treated with MF, migration was impaired by 18 h and significantly inhibited by 30 h. Whereas vehicle-treated cells nearly tripled the number of cells within the wounded area, the number of MF-treated cells in the wounded area remained similar to 0 h (Fig. 1e, f). The LNCaP prostate cancer cells were by far the slowest moving cells. However, even when they migrated at a slow pace, MF treatment still significantly attenuated migration at both 18 h and 30 h (Fig. 1g). Finally, the migration of MF-treated U87MG cells was significantly reduced, at both times studied, when compared to vehicle-treated cells (Fig. 1h).

**Fig. 2:**
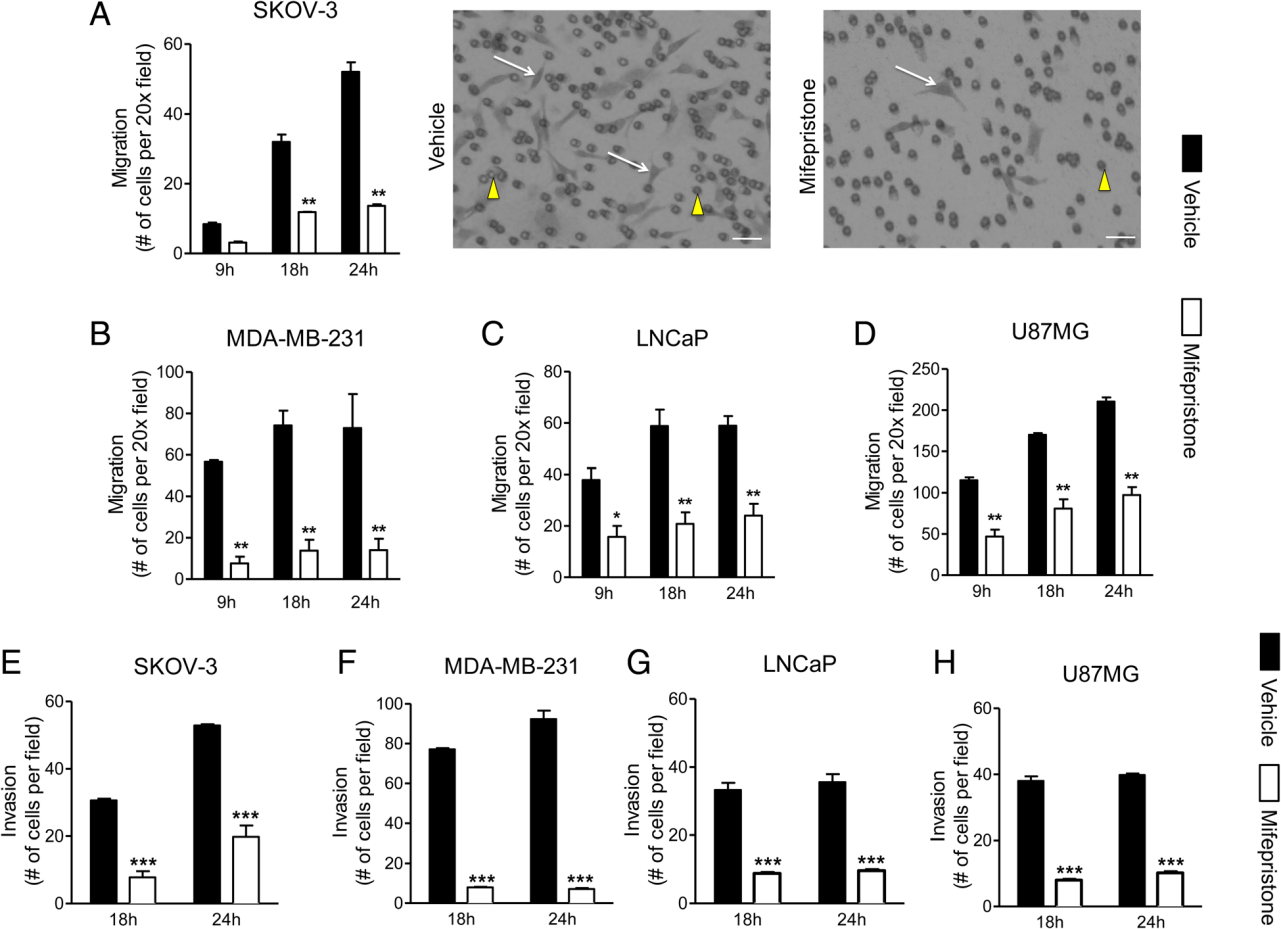
Mifepristone attenuates migration and invasion of cancer cells of the ovary (**a**, **e**), breast (**b**, **f**), prostate (**c**, **g**), or glia (**d**, **h**) through filter-based assays assessed by phase contrast microscopy. Cells were exposed to either vehicle of cytostatic concentrations of MF specific to each cell line for 72 h and then seeded into Boyden chambers. At various time-points (9, 18, and 24 h) cells that migrated through the insert were counted after staining with crystal violet. Data shown represent the mean ± s.e.m. of three independent experiments completed in triplicate (**a**-**d**). Vehicle (closed bars) and MF (open bars). Images at the right of panel A show inserts of SKOV-3 cells treated with vehicle or MF, 24 h following migration; white arrows: cells that migrated through the pores and re-attached to the other side of the membrane; yellow arrowheads: 8 μm membrane pores. Scale bars = 50 μm. *P < 0.05, **P < 0.01and ***P < 0.001 compared against vehicle. Statistical analysis was done using a two-way ANOVA followed by Bonferroni’s multiple-comparison test. For the invasion assays, data shown represent the mean ± s.e.m. of three independent experiments completed in triplicate. Vehicle (closed bars); MF (open bars)

The migration of MF-treated SKOV-3 cells through the Boyden chamber insert diminished significantly when compared to vehicle-treated cells by 18 and 24 h (Fig. 2a). The migration of MDA-MB-231 cells (Fig. 2b) and LNCaP cells (Fig. 2c) was significantly inhibited by MF pre-treatment at all evaluated time-points. U87MG cells were the most aggressive, with the largest number of cells migrating through the insert by 9 h, compared to the other cell lines. However, as early as 9 h, and at both 18 and 30-h time-points, the inhibitory effect of MF treatment was significant in comparison to vehicle-treated cells (Fig. 2d). In summary, the inhibitory effect of MF on cell migration was observed in all cell lines, regardless of their basal migratory capacity.

SKOV-3, LNCaP, and U87MG cells were similar in their invasive capacities, and MF significantly diminished the invasion of all cells after both 18 and 24 h (Fig. 2e, g, h). The breast cancer cell line, MDA-MB-231, was the most invasive, yet it also showed significant inhibition of invasion by MF at both 18 and 24 h (Fig. 2f).

### Validation of the wound healing assay in cancer cells upon MF treatment using double fluorescence staining

The inhibitory effect of MF on the migration of each cancer cell line in a wound healing assay was validated with the addition of Alexa Fluor®-594 Phalloidin and DAPI, to stain the actin cytoskeleton and nucleus, respectively. All four cell lines were studied using the same variation of the migration method.

**Fig. 3:**
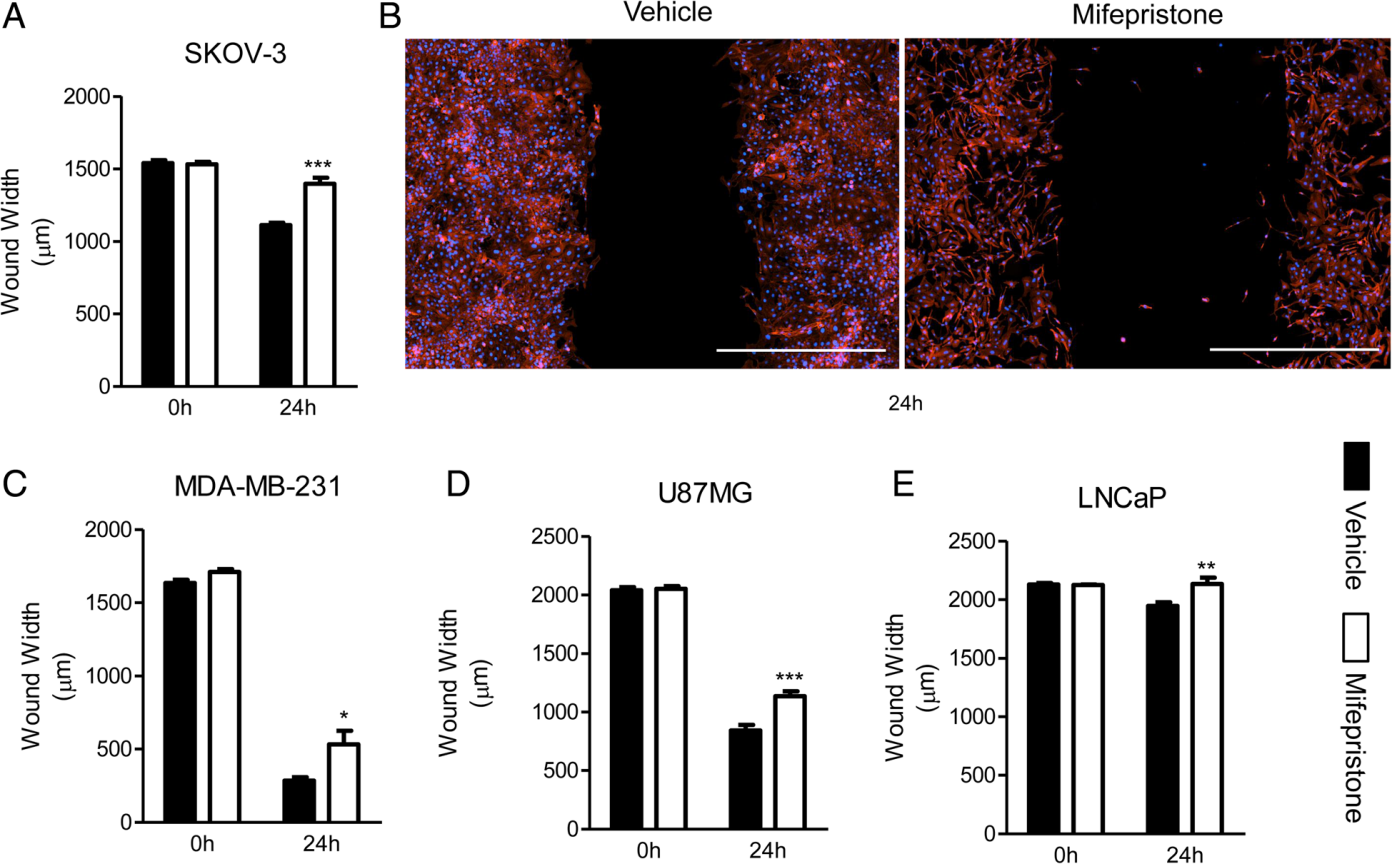
Mifepristone reduces the migration of cancer cells of the ovary (**a**, **b**), breast (**c**), glia (**d**), or prostate (**e**) in a wound healing assay using fluorescent stains. Each cell line was treated with their respective concentration of MF for 72 h prior to wounding. Multiple images were taken of each wound, and the average wound width was calculated at 0 and 24 h. The width of the wound was measured four times per image. (**b**) Is a visual representation of SKOV-3 cells in a wound healing assay, labeled with Alexa Fluor®-594 Phalloidin and DAPI, to stain the cytoskeleton and nucleus respectively. Scale bar = 1000 μm. Data in **a**, **c**, **d**, and **e** represent the mean ± s.e.m. Vehicle (closed bars); MF (open bars). *P < 0.05, **P < 0.01, ***P < 0.001 compared against vehicle. Statistical analysis was done using two-way ANOVA followed by Bonferroni’s multiple-comparison test

The migration of SKOV-3 cells was significantly reduced at 24 h. In this case, MF-treated cells barely migrated after 24 h, compared to 0 h (Fig. 3a, b). These results are consistent with the results previously described, in which MF-treated cells at 30 h were unable to move any farther than they had after 18 h (Fig. 1c). U87MG and MDA-MB-231 were the most migratory cell lines, almost completely closing the wound after 24 h; although cells treated with MF were capable of migrating, their migration rate was significantly diminished when compared to cells treated with vehicle in both cell lines (Fig. 3c, d). LNCaP was by far the slowest migrating cell line, barely closing the wound after 24 h. The average wound width of MF-treated cells was relatively the same at 0 and 24 h; MF-induced reduction of wound healing was yet found to be significant compared with untreated cells at this time point (Fig. 3e).

### Enhancement of the Boyden chamber assays in cancer cells upon MF treatment using double fluorescence staining

**Fig. 4:**
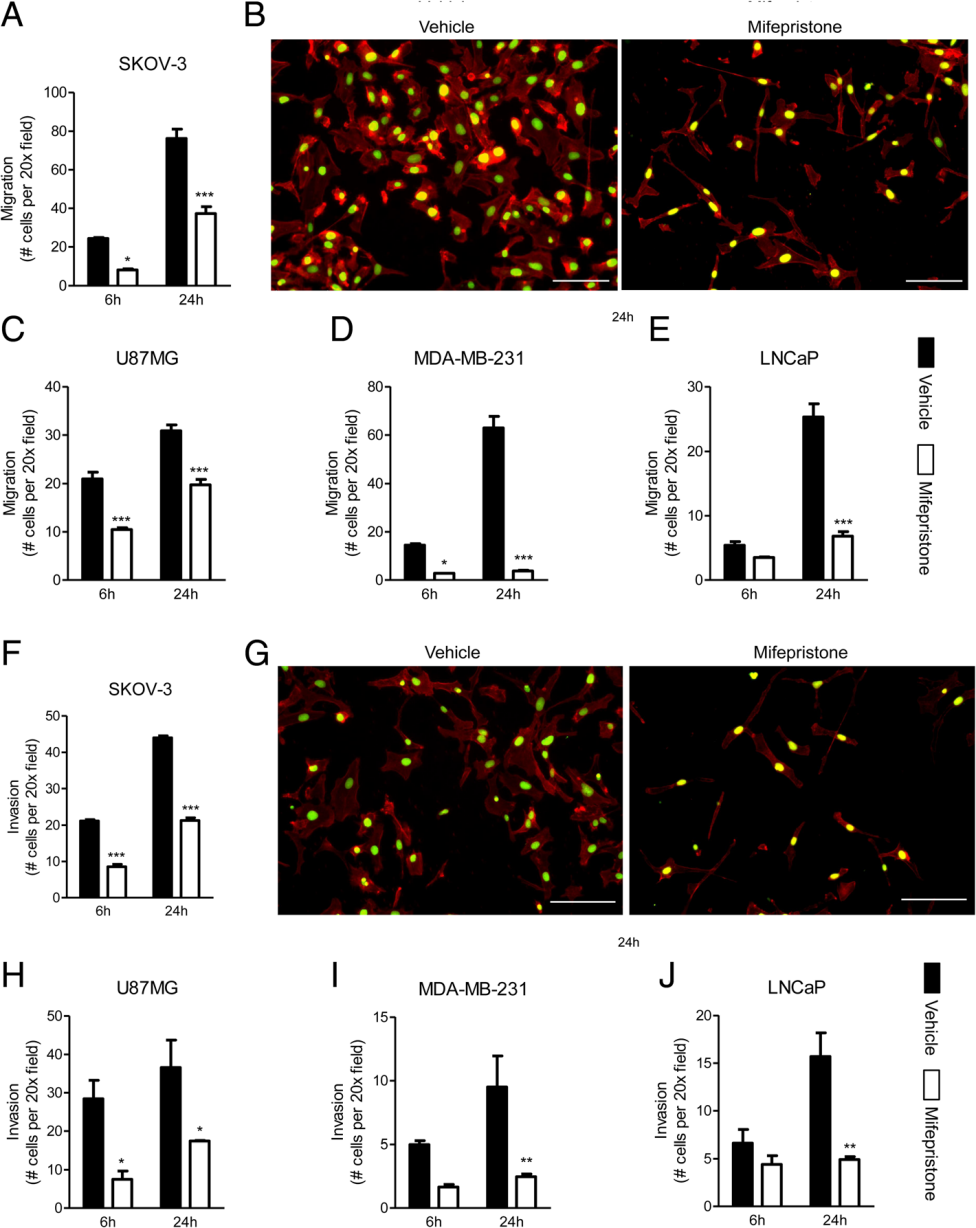
Mifepristone reduces the migration and invasion of cancer cells of the ovary (**a**, **b**, **f**, **g**), glia (**c**, **h**), breast (**d**, **i**), or prostate (**e**, **j**) in a Boyden chamber assay assessed by fluorescence microscopy. Each cell line was treated with their respective concentration of MF for 72 h prior to plating. Visual representations of migrated (**b**) and invasive (**g**) SKOV-3 cells, labeled with Alexa Fluor® 594-phalloidin and SYTOX® Green Nucleic Acid stain upon treatment with vehicle (left panels) or MF (right panels). Scale bar = 100 μm. Data shown represent the mean ± s.e.m. Vehicle (closed bars); MF (open bars). *P < 0.05, **P < 0.01, ***P < 0.001 compared against vehicle. Statistical analysis was done using two-way ANOVA followed by Bonferroni’s multiple-comparison test

Upon enhancement of the staining of migratory cells using Alexa Fluor®-594 Phalloidin and SYTOX® Green, the effect of MF remained consistent in all cell lines. SKOV-3 cells were found to be the most migratory and demonstrated a large increase in the number of migrated cells between 6 and 24 h. MF was able to significantly inhibit the migration of these cells as early as 6 h after the start of the experiment (Fig. 4a, b). U87MG and MDA-MB-231 cells were once again found to be highly migratory through this assay, although these experiments showed MDA-MD-231 cells to be more migratory than U87MG cells. In both cases, cells were found to be quite migratory at 6 h, however MDA-MB-231 demonstrated a much larger increase in the number of migratory cells at 24 h compared to U87MG. In both cell lines, MF was able to attenuate the migration at 6 and 24 h, however this effect was most drastically observed in MDA-MB-231, where cells were unable to migrate more than they had after 6 h, even after 24 h (Fig. 4c, d). Finally, LNCaP was once again the slowest migrating cell line. In this case, similar to MDA-MB-231, at 6 h, MF-treated cells were unable to migrate any farther than they had at 24 h; however, this inhibition was only significant when compared to vehicle-treated cells at 24 h (Fig. 4e).

To study and validate the invasion and the effect of MF on each cell line, the Boyden chamber assay was repeated but with the addition of a layer of ECM in the upper chamber. Once again, the effect of MF was consistent. SKOV-3 and U87MG had a similar invasive rate, and in both cell lines, the inhibitory effect of MF was observed after 6 and 24 h (Fig. 4f-h). MDA-MB-231 and LNCaP cells both had a slower invasive capacity. However, MF was still able to significantly inhibit their invasiveness (Fig. 4i-j).

### The addition of double fluorescence staining to migration assays unveils varying migration patterns in cancer cells

A crucial event of cancer metastasis is the migration of cancer cells from the primary tumor to secondary, distant sites. Two distinct patterns of migration have been described: single-cell and collective-cell migration (rev. in [17–20]). In single-cell migration, cells migrate individually and invade surrounding tissues independent of each other. Collective-cell migration involves groups of cells adherent to one another, migrating and invading surrounding tissues as multicellular aggregates. By enhancing the wound healing and Boyden chamber assays with the addition of a double fluorescence staining—labeling actin cytoskeleton and nuclear DNA—it is possible to better observe such migration patterns.

**Fig. 5:**
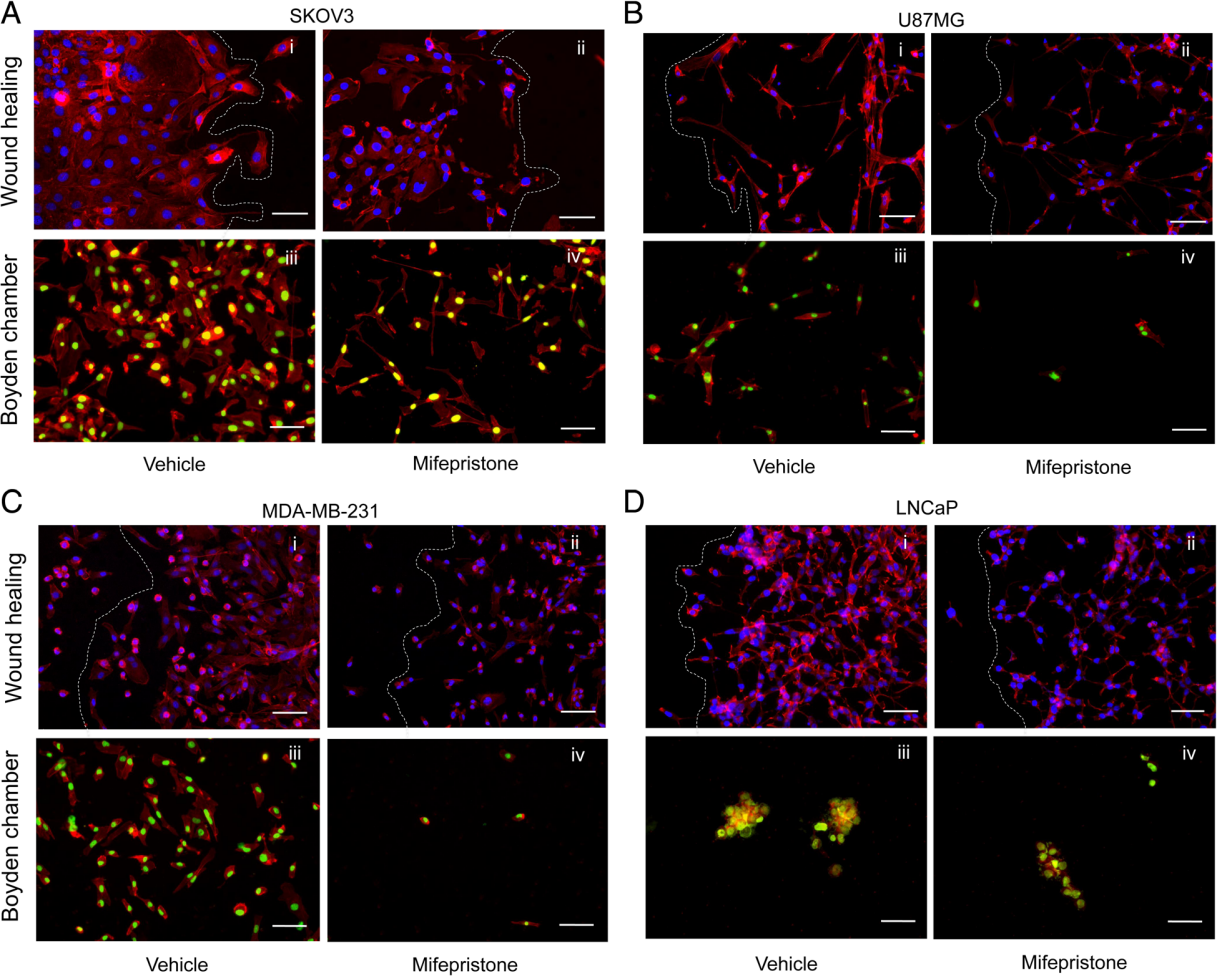
Enhancing the wound healing and Boyden chamber assays with a double fluorescence labeling allows for the visualization of different migration patterns between cell lines. SKOV-3 (**a**), U87MG (**b**), MDA-MB-231 (**c**), or LNCaP (**d**) were all treated with their respective concentrations of MF for 72 h. A wound healing assay and a Boyden chamber assay were performed for each cell line. After 24 h, cells were fixed with 4% PFA, and stained with Alexa Fluor®-594 Phalloidin and DAPI (wound healing), or SYTOX® Green Nucleic Acid stain (Boyden chamber). Scale bars = 75 μm. White lines represent the front of the wound

When observing SKOV-3 cells in a wound healing assay, it was noticed that cells treated with vehicle formed a sheet as they closed the wound, suggesting collective-cell migration (Fig. 5a, i; and Fig. 3b). SKOV-3 cells having migrated through the pores in the Boyden chamber seemed to recreate this phenomenon, as cells were observed to be covering the entire surface of the image, almost always touching one another (Fig. 5a, iii). When SKOV-3 cells were treated with MF, the cells in the front leading edge were more isolated and less adherent to one another. This change in organization can be visualized through both wound healing and Boyden Chamber methods (Fig. 5a, ii and iv; and Fig. 3b). In both wound healing and Boyden chamber migratory assays, U87MG and MDA-MB-231 were observed to express a similar migration pattern, with vehicle-treated cells loosely organized and with very little attachment to one another (Fig. 5b, i and iii; and c, i and iii). This behavior indicates that these cancer cell lines most likely undergo single-cell migration. When both U87MG and MDA-MB-231 cells were treated with MF, although the pattern remained similar to that of vehicle-treated cells, there was an obvious decrease in the number of migratory cells (Fig. 5b, ii and iv; and ,c ii and iv). Finally, LNCaP cells demonstrated a mixed migration pattern (Fig. 5d, i and iii). In the wound healing assay, some LNCaP leading cells show a mix of collective and individual migration (Fig. 5d,i). The collective migration was more obvious when the cells traversed the pores of the polycarbonate membrane of the Boyden chamber and appear as tri-dimensional clusters (Fig. 5d, iii). When LNCaP cells were treated with MF, in the wound healing assay, cells were observed to be less cohesive; yet, the front leading edge retained grouped cells (Fig. 5d, ii). Such grouping or clustering was also maintained when cells migrated through the pores of the Boyden chamber (Fig. 5d, iv).

## Discussion

In this work we focused on studying whether MF operating at cytostatic doses, while altering the morphology of the cytoskeleton and reducing cellular adhesion, also negatively impacts the migratory and invasive capacities of the cancer cells. Furthermore, we validated these approaches using an enhanced version of standard migration and invasion assays adding an extra, yet simple, visualization step involving double fluorescence staining of the cytoskeleton and the DNA, upon cytochemical reactions.

The use of fluorescent labeling of the cells to assess their migration and invasion capacities is not novel. However, these methods usually rely on the in vivo labeling of the cells with vital fluorescent stains that are retained by the cells for a number of hours [5, 21], adding the confounding factor that another chemical is added to the cells while assessing mobility. In our work, we stained the cells with fluorescent stains post-fixation, providing certainty that the staining did not influence migration/invasion. Another advantage of the method is that it does not involve an immune reaction: it is based on the affinity of Phalloidin to F-actin, and of SYTOX® Green or DAPI to DNA, making the approach easy to perform, robust, and straightforward. Finally, the double staining provides extra-information on the morphology of the cytoskeleton while cells are migrating under different conditions. The addition of fluorescence labeling uncovered varying migration patterns between different cancer cell lines. Certain cell lines were found to undergo single-cell migration, while others were found to undergo collective-cell migration (rev. in [17–19]). Notably, staining F-actin and DNA allowed the distinction of these two types of migratory patterns, as well as the recognition of cell lines with a mixed pattern of migration.

Moreover, in the assays involving movement through a membrane, the double staining allows for detailed visualization of how cellular compartments move through the 8 μm membrane pores, giving precise information on the localization of the nucleus relative to the movement of the cytoplasm. This is important as the positioning and movement of the nuclei are essential for the process of migration and, hence, invasion [22]. For instance, whereas in bi-dimensional models the nucleus is often positioned in the back of the cell, during tri-dimensional migration the nucleus may be positioned in the front or back of the cell depending on cellular type (rev. in [23]) (Additional file 2: Figure S2). During metastasis, the nucleus leading edge is the main barrier when migrating through tight spaces, such as when cancer cells pass through an endothelial cell layer. Supporting this concept, it was demonstrated with leucocytes that the localization of their nuclear lobes at the forefront of the cells act as a ‘drilling’ device by bending endothelial actin [24].

Finally, another feature that was enhanced using dual fluorescence along tri-dimensional migration (Boyden chamber assay) was the capacity to detect modifications in the distribution of F-actin among the cytoplasm and the nucleus. This was evident in SKOV-3 and LNCaP cells in which treatment with MF increased the number of cells showing yellow fluorescence in the nucleus, signifying an overlapping of SYTOX® Green Nucleic Acid Stain binding DNA (green) with that of AlexaFluor®594 Phalloidin binding F-actin (red) (Additional file 3: Figure S3). This feature is important to point out as F-actin can be visualized in the cytoplasm and the nucleus under particular conditions as a result of its movement via transport molecules (rev. in [25]). For instance, nuclear actin has been shown to be important in chromatin remodeling and organization [26], and during cell death [27], highlighting the relevance of its location, in particular, when exposing cells to cytotoxic agents.

Expectations suggest that the impairment in the adhesive capacity of cells exposed to MF should negatively impact their dissemination capacity, because both de-adhesion and adhesion are critical to cancer cell metastasis, since they reflect the initial detachment from the primary tumor site and the re-attachment leading to re-growth at a secondary location. In addition, cell migration is characterized by cyclic detachments of the rear of the cells and attachments to the front, which propel the cell forward. The turnover rate of these adhesions and de-adhesions is critical for migration, and a relationship has been shown between cell de-adhesion and rate of migration [28]. We previously demonstrated that when cancer cells are exposed to cytostatic concentrations of MF, they suffer significant decrease of their ability to attach to various extracellular matrix proteins while suffering major alterations in the distribution of F-actin associated with an increase in the formation of actin ruffles with no adhesion capacity [13]. These results were recently confirmed in two ovarian cancer cells lines in which MF caused a decrease in the visualization of stress actin fibers, with a concomitant increase in cortical actin and reduced adhesion to extracellular matrix proteins [29]. MF also retained the anti-adhesive properties against human melanoma cells when given in combination with doxycycline, aspirin, and lysine [30]. Furthermore, it was demonstrated that the major metabolite of MF, a mono-demethylated derivative termed metapristone [31], is capable of diminishing adhesion of HT-29 colorectal adenocarcinoma cells to human extracellular matrix and to umbilical vein endothelial cells (HUVECs) [32].

The effect of MF reducing cellular adhesion and changing cellular morphology, from their original epithelial shape towards a spindle-like appearance, suggests that the speed of migration may also be positively affected, likely involving EMT [33]; however, contrary to the expectation, in all cancer cells studied, treatment with MF significantly attenuated migration of cells in wound healing assays. Firstly, we validated the results of the wound healing assay by showing that cells transiting M phase seem to only be found away from the wound, but not at the wound site, as demonstrated by the expression of the mitotic marker pHH3 (Additional file 4: Figure S4) [34, 35]. As mitoses are usually coupled to cell proliferation [36], labeling with pHH3 could prevent the need to use serum starvation or mitotic poisons to block cell division without killing the cells and, at the same time, allows closure of the wound to be attributed to migration and not cell proliferation [21, 37]. Secondly, we validated the negative action of MF on migration in a tri-dimensional (Boyden chamber) assay. MF was capable of diminishing cell migration through the 8 μm pore polycarbonate membrane as early as 6 h in each cell line studied. Supporting our data with ovarian, breast, glial, and prostate cancer cell lines, it was reported very recently that MF inhibited migration induced by progesterone in human astrocytoma cells [38], that both MF and its metabolite metapristone inhibited the chemotactic migration and mobility in SKOV-3 and IGROV-1 ovarian cancer cell lines facilitated by activation of the chemokine SDF-1/CXCR4 [29, 39], and that MF inhibited migration and invasion of endometrial carcinoma cells [40].

MF may have the simultaneous ability to inhibit cell growth and migration via a common mechanism: increase in expression of cyclin-dependent kinase inhibitor p21^cip1^. We have shown that MF and MF-related compounds block growth of cancer cells inhibiting the activity of cyclin-dependent kinase Cdk2 as a consequence of p21^cip1^ upregulation [9–11]. In support of this concept, it was found that inducing p21^cip1^ expression inhibits vascular smooth muscle cell proliferation and migration [41]. More studies need to be done addressing the role of p21^cip1^ in cancer-cell migration; however, the possibility of MF carrying out its various effects through cell-cycle inhibitors should not be overlooked.

Another in vitro aspect studied was cellular invasion, which involves the penetration through tissue barriers, including the basement membrane and stroma, and involves adhesion, proteolytic degradation of the extracellular matrix, and migration [28]. We clearly showed previously that MF treatment significantly reduced adherence [13], and now demonstrate that it also attenuates migration and invasion of four highly metastatic cancer cell lines. Supporting our results, in astrocytoma cells, MF blocked the acceleration in the invasive capacity of the cells triggered by progesterone [38]. In addition, in human gastric adenocarcinoma cells, MF inhibited adhesion, migration, and invasion [42]. Also, in two melanoma cell lines, metapristone, the demethylated metabolite of MF [31], significantly impaired invasion through the complex extracellular matrix contained in Matrigel® [43].

## Conclusions

Our results clearly show that MF can successfully impair the machinery necessary for cellular migration and invasion, while simultaneously inhibiting cell proliferation. Since cell migration and invasion are major components of metastasis, it can be inferred that usage of MF may indeed prove to be part of a successful anti-metastatic therapy. Furthermore, we used the anti-migratory and anti-invasive actions of MF to demonstrate that migration and invasion assays can be advanced in terms of their visualization by using double fluorescence cytochemical staining of cytoskeletal fibers and DNA.

## Additional files

Additional file 1: **Figure S1.** Alterations in cellular morphology caused by cytostatic concentrations of MF in cancer cells visualized using fluorescent stains. (**A**) SKOV-3, (**B**) MDA-MB-231, (**C**) LNCaP, or (**D**) U87MG cells were plated at a density of 100,000 cells/well for both vehicle and MF-treated groups, and allowed to attach overnight. Treatment with increasing concentrations of the drug (0, vehicle; 5, 5 μM MF; 10, 10 μM MF; 20, 20 μM MF; 30, 30 μM MF; and 40, 40 μM MF) was provided for 72 h. Cells were then fixed with 4% PFA and stained with AlexaFluor®594-phalloidin and SYTOX®Green Nucleic Acid Stain. Scale bars = 20 μm. (TIF 8971 kb)

Additional file 2: **Figure S2.** Enhancing the wound healing assay (2D migration assay) and the Boyden chamber assay (3D migration assay) with a double fluorescence labelling allows for the visualization of the position of the nucleus relative to the cytoplasm in migrating cells. U87MG (**A**) and SKOV-3 (**B**) were subjected to migration in a wound healing assay. MDA-MB-231 (**C**) and LNCaP (**D**) were subjected to migration in a Boyden chamber assay. Arrows, nucleus at the back of the cell; arrowheads, nuclei at the front of the cell. White lines in A and B mark the border of the wound. Scale bars = 75 μm. (TIF 6039 kb)

Additional file 3: **Figure S3.** Observing relative distribution of F-actin within nucleus and cytoplasm. Images depict migration through a Boyden chamber of SKOV-3 or LNCaP cells receiving vehicle (**A** and **C**) or MF (**B** and **D**). Large white arrows denote nuclei stained in yellow, signifying that staining for F-actin seems to be increasing when compared against nuclei seen in green. In this case, treatment with MF, while diminishing the number of migrating cells, seems to increase the number of such cells having increased F-actin in their nuclei. Scale bars = 90 μm. (TIF 3633 kb)

Additional file 4: **Figure S4.** Cells closer to the wound express little to no pHH3 when compared with cells located farther away from the wound. SKOV-3 (**A**, **B**, **E**, **F**) and U87MG (**C**, **D**, **G**, **H**) were treated with their respective concentrations of MF for 72 h. A wound healing assay was then performed as described in materials and methods. After 24 h, cells were fixed with 4% PFA and labeled for pHH3 through immunocytochemistry with the addition of Alexa Fluor® 594-phalloidin to stain the cytoplasm. Scale bar = 75 μm. White lines in **A**, **B**, **C**, and **D** represent the border of the wound. (TIF 8846 kb)

## Abbreviations

ANOVA: Analysis of Variance
BSA: Bovine Serum Albumin
CXCR4: C-X-C chemokine receptor type 4
EMT: Epithelial Mesenchymal Transition
F-actin: Fibrillar actin
FBS: Fetal Bovine Serum
MF: mifepristone
PBS: Phosphate Buffered Saline
pHH3: Phospho-Histone H3
SDF-1: Stromal derived factor-1

## References

[1] Steeg PS (2006). Tumor metastasis: mechanistic insights and clinical challenges. Nat Med.

[2] Le Devedec SE, Yan K, de Bont H, Ghotra V, Truong H, Danen EH, Verbeek F, van de Water B (2010). Systems microscopy approaches to understand cancer cell migration and metastasis. Cell Mol Life Sci.

[3] Vignjevic D, Montagnac G (2008). Reorganisation of the dendritic actin network during cancer cell migration and invasion. Semin Cancer Biol.

[4] Valastyan S, Weinberg RA (2011). Tumor metastasis: molecular insights and evolving paradigms. Cell.

[5] Eccles SA, Box C, Court W (2005). Cell migration/invasion assays and their application in cancer drug discovery. Biotechnol Annu Rev.

[6] Chabner BA, Roberts TG (2005). Timeline: chemotherapy and the war on cancer. Nat Rev Cancer.

[7] Huang M, Shen A, Ding J, Geng M (2014). Molecularly targeted cancer therapy: some lessons from the past decade. Trends Pharmacol Sci.

[8] Pardoll DM (2012). The blockade of immune checkpoints in cancer immunotherapy. Nat Rev Cancer.

[9] Tieszen CR, Goyeneche AA, Brandhagen BN, Ortbahn CT, Telleria CM (2011). Antiprogestin mifepristone inhibits the growth of cancer cells of reproductive and non-reproductive origin regardless of progesterone receptor expression. BMC Cancer.

[10] Goyeneche AA, Seidel EE, Telleria CM (2012). Growth inhibition induced by antiprogestins RU-38486, ORG-31710, and CDB-2914 in ovarian cancer cells involves inhibition of cyclin dependent kinase 2. Investig New Drugs.

[11] Goyeneche AA, Caron RW, Telleria CM (2007). Mifepristone inhibits ovarian cancer cell growth in vitro and in vivo. Clin Cancer Res.

[12] Zhang L, Hapon MB, Goyeneche AA, Srinivasan R, Gamarra-Luques CD, Callegari EA, Drappeau DD, Terpstra EJ, Pan B, Knapp JR (2016). Mifepristone increases mRNA translation rate, triggers the unfolded protein response, increases autophagic flux, and kills ovarian cancer cells in combination with proteasome or lysosome inhibitors. Mol Oncol.

[13] Brandhagen BN, Tieszen CR, Ulmer TM, Tracy MS, Goyeneche AA, Telleria CM (2013). Cytostasis and morphological changes induced by mifepristone in human metastatic cancer cells involve cytoskeletal filamentous actin reorganization and impairment of cell adhesion dynamics. BMC Cancer.

[14] Chhabra ES, Higgs HN (2007). The many faces of actin: matching assembly factors with cellular structures. Nat Cell Biol.

[15] Jonkman JE, Cathcart JA, Xu F, Bartolini ME, Amon JE, Stevens KM, Colarusso P (2014). An introduction to the wound healing assay using live-cell microscopy. Cell Adhes Migr.

[16] Poujade M, Grasland-Mongrain E, Hertzog A, Jouanneau J, Chavrier P, Ladoux B, Buguin A, Silberzan P (2007). Collective migration of an epithelial monolayer in response to a model wound. Proc Natl Acad Sci U S A.

[17] De Pascalis C, Etienne-Manneville S (2017). Single and collective cell migration: the mechanics of adhesions. Mol Biol Cell.

[18] Ilina O, Friedl P (2009). Mechanisms of collective cell migration at a glance. J Cell Sci.

[19] Krakhmal NV, Zavyalova MV, Denisov EV, Vtorushin SV, Perelmuter VM (2015). Cancer invasion: patterns and mechanisms. Acta Nat.

[20] Collins C, Nelson WJ (2015). Running with neighbors: coordinating cell migration and cell-cell adhesion. Curr Opin Cell Biol.

[21] Glenn HL, Messner J, Meldrum DR (2016). A simple non-perturbing cell migration assay insensitive to proliferation effects. Sci Rep.

[22] Gundersen GG, Worman HJ (2013). Nuclear positioning. Cell.

[23] Calero-Cuenca FJ, Janota CS, Gomes ER (2018). Dealing with the nucleus during cell migration. Curr Opin Cell Biol.

[24] Barzilai S, Yadav SK, Morrell S, Roncato F, Klein E, Stoler-Barak L, Golani O, Feigelson SW, Zemel A, Nourshargh S (2017). Leukocytes breach endothelial barriers by insertion of nuclear lobes and disassembly of endothelial actin filaments. Cell Rep.

[25] Izdebska M, Zielinska W, Grzanka D, Gagat M (2018). The role of actin dynamics and actin-binding proteins expression in epithelial-to-mesenchymal transition and its association with Cancer progression and evaluation of possible therapeutic targets. Biomed Res Int.

[26] Castano E, Philimonenko VV, Kahle M, Fukalova J, Kalendova A, Yildirim S, Dzijak R, Dingova-Krasna H, Hozak P (2010). Actin complexes in the cell nucleus: new stones in an old field. Histochem Cell Biol.

[27] Grzanka D, Gagat M, Izdebska M (2014). Involvement of the SATB1/F-actin complex in chromatin reorganization during active cell death. Int J Mol Med.

[28] Friedl P, Wolf K (2003). Tumour-cell invasion and migration: diversity and escape mechanisms. Nat Rev Cancer.

[29] Zheng N, Chen J, Liu W, Liu J, Li T, Chen H, Wang J, Jia L (2017). Mifepristone inhibits ovarian cancer metastasis by intervening in SDF-1/CXCR4 chemokine axis. Oncotarget.

[30] Wan L, Dong H, Xu H, Ma J, Zhu Y, Lu Y, Wang J, Zhang T, Li T, Xie J (2015). Aspirin, lysine, mifepristone and doxycycline combined can effectively and safely prevent and treat cancer metastasis: prevent seeds from gemmating on soil. Oncotarget.

[31] Heikinheimo O, Kekkonen R, Lahteenmaki P (2003). The pharmacokinetics of mifepristone in humans reveal insights into differential mechanisms of antiprogestin action. Contraception.

[32] Wang J, Chen J, Wan L, Shao J, Lu Y, Zhu Y, Ou M, Yu S, Chen H, Jia L (2014). Synthesis, spectral characterization, and in vitro cellular activities of metapristone, a potential cancer metastatic chemopreventive agent derived from mifepristone (RU486). AAPS J.

[33] Morris HT, Machesky LM (2015). Actin cytoskeletal control during epithelial to mesenchymal transition: focus on the pancreas and intestinal tract. Br J Cancer.

[34] Hendzel MJ, Wei Y, Mancini MA, Van Hooser A, Ranalli T, Brinkley BR, Bazett-Jones DP, Allis CD (1997). Mitosis-specific phosphorylation of histone H3 initiates primarily within pericentromeric heterochromatin during G2 and spreads in an ordered fashion coincident with mitotic chromosome condensation. Chromosoma.

[35] Tsuta K, Liu DC, Kalhor N, Wistuba II, Moran CA (2011). Using the mitosis-specific marker anti-phosphohistone H3 to assess mitosis in pulmonary neuroendocrine carcinomas. Am J Clin Pathol.

[36] Batistatou A (2004). Mitoses and cancer. Med Hypotheses.

[37] Reinhart-King CA (2008). Endothelial cell adhesion and migration. Methods Enzymol.

[38] Pina-Medina AG, Hansberg-Pastor V, Gonzalez-Arenas A, Cerbon M, Camacho-Arroyo I (2016). Progesterone promotes cell migration, invasion and cofilin activation in human astrocytoma cells. Steroids.

[39] Zheng N, Chen J, Li T, Liu W, Liu J, Chen H, Wang J, Jia L (2017). Abortifacient metapristone (RU486 derivative) interrupts CXCL12/CXCR4 axis for ovarian metastatic chemoprevention. Mol Carcinog.

[40] Sang L, Lu D, Zhang J, Du S, Zhao X (2018). Mifepristone inhibits proliferation, migration and invasion of HUUA cells and promotes its apoptosis by regulation of FAK and PI3K/AKT signaling pathway. Onco Targets Ther.

[41] Fukui R, Shibata N, Kohbayashi E, Amakawa M, Furutama D, Hoshiga M, Negoro N, Nakakouji T, Ii M, Ishihara T (1997). Inhibition of smooth muscle cell migration by the p21 cyclin-dependent kinase inhibitor (Cip1). Atherosclerosis.

[42] Li DQ, Wang ZB, Bai J, Zhao J, Wang Y, Hu K, Du YH (2004). Effects of mifepristone on invasive and metastatic potential of human gastric adenocarcinoma cell line MKN-45 in vitro and in vivo. World J Gastroenterol.

[43] Zheng N, Chen J, Liu W, Wang J, Liu J, Jia L (2017). Metapristone (RU486 derivative) inhibits cell proliferation and migration as melanoma metastatic chemopreventive agent. Biomed Pharmacother.

