# Supplementary figures and images for: Advanced assessment of migration and invasion of cancer cells in response to mifepristone therapy using double fluorescence cytochemical labeling
Source: BMC Cancer. 2019 Apr 24;19:376. doi: 10.1186/s12885-019-5587-3 (PMC6480622; doi:10.1186/s12885-019-5587-3)

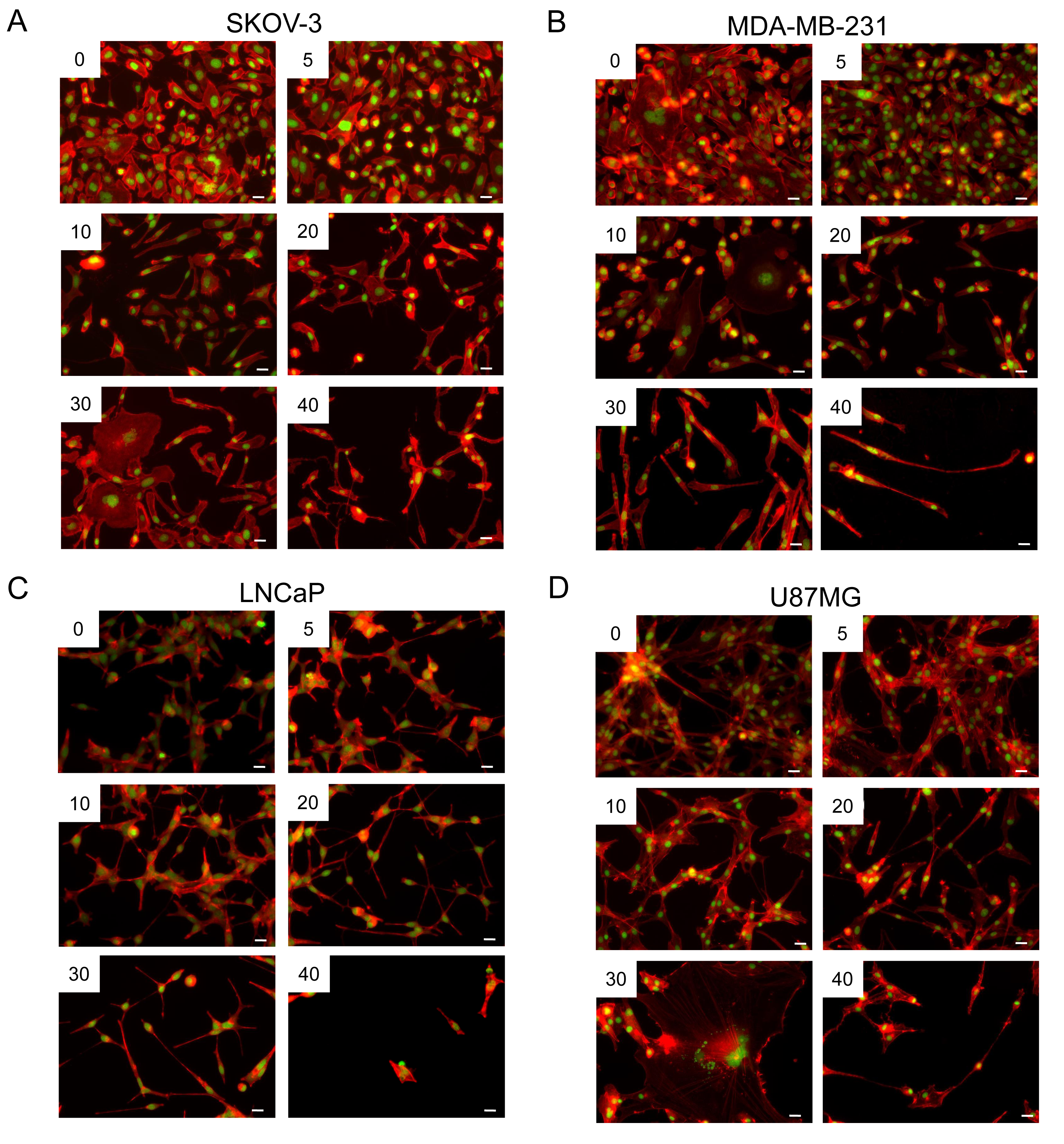

Supplement: Supplementary file 1 — Figure S1. Alterations in cellular morphology caused by cytostatic concentrations of MF in cancer cells visualized using fluorescent stains. (A) SKOV-3, (B) MDA-MB-231, (C) LNCaP, or (D) U87MG cells were plated at a density of 100,000 cells/well for both vehicle and MF-treated groups, and allowed to attach overnight. Treatment with increasing concentrations of the drug (0, vehicle; 5, 5 μM MF; 10, 10 μM MF; 20, 20 μM MF; 30, 30 μM MF; and 40, 40 μM MF) was provided for 72 h. Cells were then fixed with 4% PFA and stained with AlexaFluor®594-phalloidin and SYTOX®Green Nucleic Acid Stain. Scale bars = 20 μm. (TIF 8971 kb) [file 12885_2019_5587_MOESM1_ESM.tif]

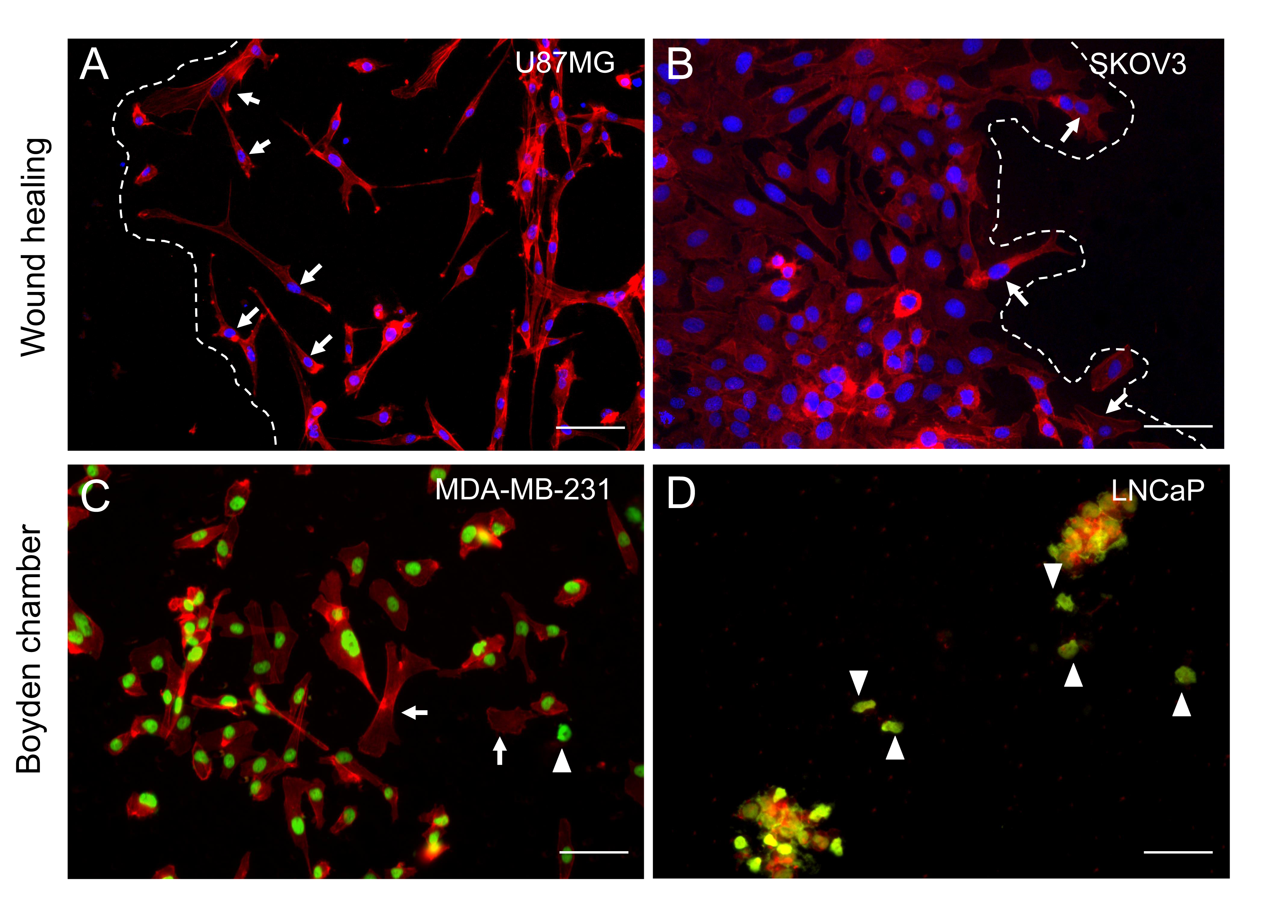

Supplement: Supplementary file 2 — Figure S2. Enhancing the wound healing assay (2D migration assay) and the Boyden chamber assay (3D migration assay) with a double fluorescence labelling allows for the visualization of the position of the nucleus relative to the cytoplasm in migrating cells. U87MG (A) and SKOV-3 (B) were subjected to migration in a wound healing assay. MDA-MB-231 (C) and LNCaP (D) were subjected to migration in a Boyden chamber assay. Arrows, nucleus at the back of the cell; arrowheads, nuclei at the front of the cell. White lines in A and B mark the border of the wound. Scale bars = 75 μm. (TIF 6039 kb) [file 12885_2019_5587_MOESM2_ESM.tif]

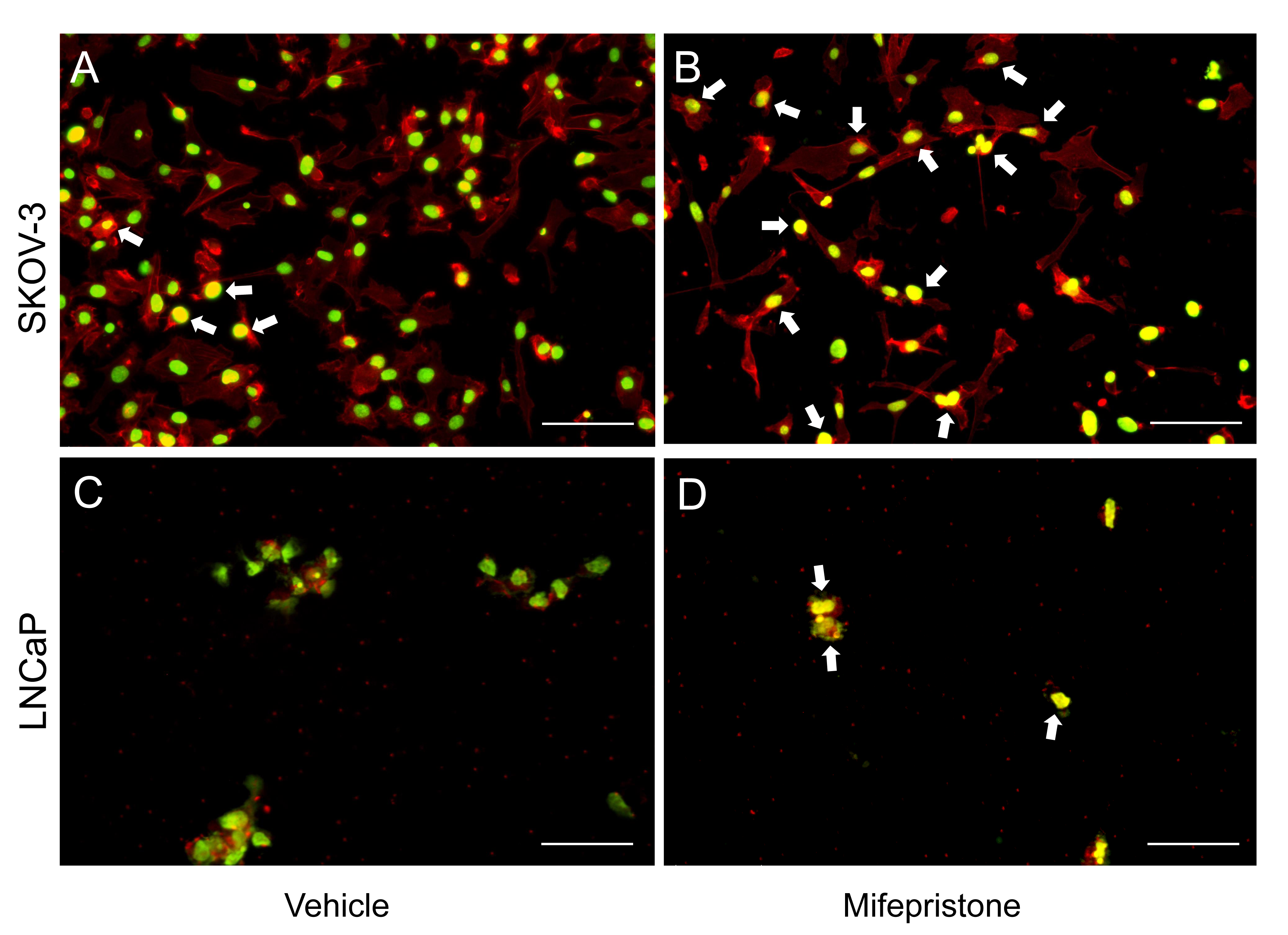

Supplement: Supplementary file 3 — Figure S3. Observing relative distribution of F-actin within nucleus and cytoplasm. Images depict migration through a Boyden chamber of SKOV-3 or LNCaP cells receiving vehicle (A and C) or MF (B and D). Large white arrows denote nuclei stained in yellow, signifying that staining for F-actin seems to be increasing when compared against nuclei seen in green. In this case, treatment with MF, while diminishing the number of migrating cells, seems to increase the number of such cells having increased F-actin in their nuclei. Scale bars = 90 μm. (TIF 3633 kb) [file 12885_2019_5587_MOESM3_ESM.tif]

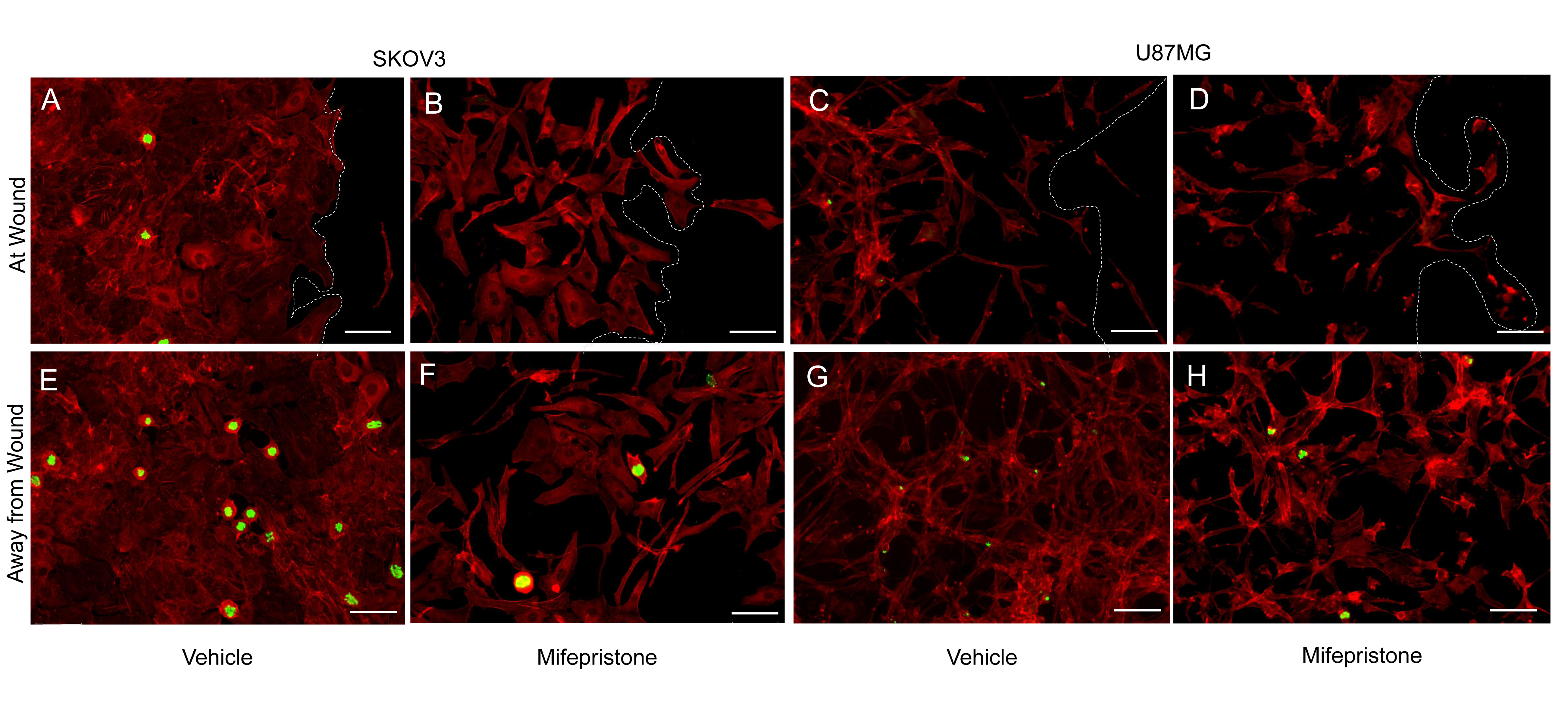

Supplement: Supplementary file 4 — Figure S4. Cells closer to the wound express little to no pHH3 when compared with cells located farther away from the wound. SKOV-3 (A, B, E, F) and U87MG (C, D, G, H) were treated with their respective concentrations of MF for 72 h. A wound healing assay was then performed as described in materials and methods. After 24 h, cells were fixed with 4% PFA and labeled for pHH3 through immunocytochemistry with the addition of Alexa Fluor® 594-phalloidin to stain the cytoplasm. Scale bar = 75 μm. White lines in A, B, C, and D represent the border of the wound. (TIF 8846 kb) [file 12885_2019_5587_MOESM4_ESM.tif]
